# Counseling patients with higher-risk MDS regarding survival with azacitidine therapy: are we using realistic estimates?

**DOI:** 10.1038/s41408-018-0081-8

**Published:** 2018-06-11

**Authors:** Amer M. Zeidan, Maximilian Stahl, Michelle DeVeaux, Smith Giri, Scott Huntington, Nikolai Podoltsev, Rong Wang, Xiaomei Ma, Amy J. Davidoff, Steven D. Gore

**Affiliations:** 1 Yale University and Yale Cancer Center, New Haven, CT USA; 2 Yale Cancer Outcomes Public Policy, and Effectiveness Research (COPPER) Center, New Haven, CT USA; 3 Department of Biostatistics, Yale School of Public Health, New Haven, CT USA; 4 Department of Chronic Diseases, Yale School of Public Health, Yale University, New Haven, CT USA; 5 Department of Health Policy and Management, School of Public Health, Yale University, New Haven, CT USA

Azacitidine is the only drug proven to prolong overall survival (OS) in patients with higher-risk myelodysplastic syndromes (HR-MDS) in the large randomized trial AZA-001 with a median OS of 24.5 months among azacitidine-treated patients vs. 15.0 months in the conventional care regimens (CCR) arm, with a hazard ratio of 0.58; *p* = 0.0001^1^. Two-year OS probability was 51% in azacitidine arm compared with 26% in the CCR arm (*p* < 0.0001). These numbers are often quoted to counsel HR-MDS patients regarding expected benefits with azacitidine therapy.

However, several real-life data and registry studies suggest that the median OS benefit with azacitidine is much lower (a median of 13–16 months) than what is suggested by AZA-001 trial^2–8^. Additionally, in a recent large Surveillance, Epidemiology, and End Results (SEER)-Medicare-linked database study of 532 patients with refractory anemia and excessive blasts (RAEB), the median OS was found to be only 11 months [95% confidence interval (CI), 10–14 months] for azacitidine treated patients in the United States (US)^9^. Furthermore, comparing OS for all patients with MDS before the approval of azacitidine and decitabine (2001–2003) and after their approval (2007–2010) using SEER Medicare data shows that the OS for MDS patients has not improved substantially after the approval of the two hypomethylating agents azacitidine and decitabine in 2004 and 2006, respectively, in the US^10^. When analysis was restricted to patients with RAEB, a proxy for HR-MDS, an improvement in OS of only 3 months was observed, which is quite small compared with the initial survival advantage of 9.5 months reported in the AZA-001 trial. Similarly, an analysis of 1000 MDS patients at the Mayo Clinic showed that OS has not significantly improved for patients with MDS over the last two decades^8^.

Certainly, the marked difference in median OS between the AZA-001 trial and real-life analyses can be attributed to many factors including the stricter selection of patients in clinical trials compared to registry studies, which often include older and frail patients. To get a more realistic estimate of OS with azacitidine and minimize the effect of selection bias when comparing clinical trial data with real-life data, we pooled OS data from these clinical trials including the landmark AZA-001 trial. First, we conducted a literature search for published prospective clinical trials that had an azacitidine monotherapy arm at the standard approved dose (75 mg/m^2^/day for 7 days) in which OS results were presented in Kaplan–Meier (KM) methodology. Next, we used GetData Graph Digitizer Version 2.26 to digitize the published KM curves of the azacitidine monotherapy arms in these trials. An algorithm developed by Guyot et al. was implemented in the R statistical software to recreate individual patient level data based on the information from each KM curve, number of patients at risk, and number of events^11^. Individual patient level data were pooled for patients receiving azacitidine to produce overall KM estimates and estimates of median OS and 1-, 2- and 3-year OS probabilities.

**Fig. 1:**
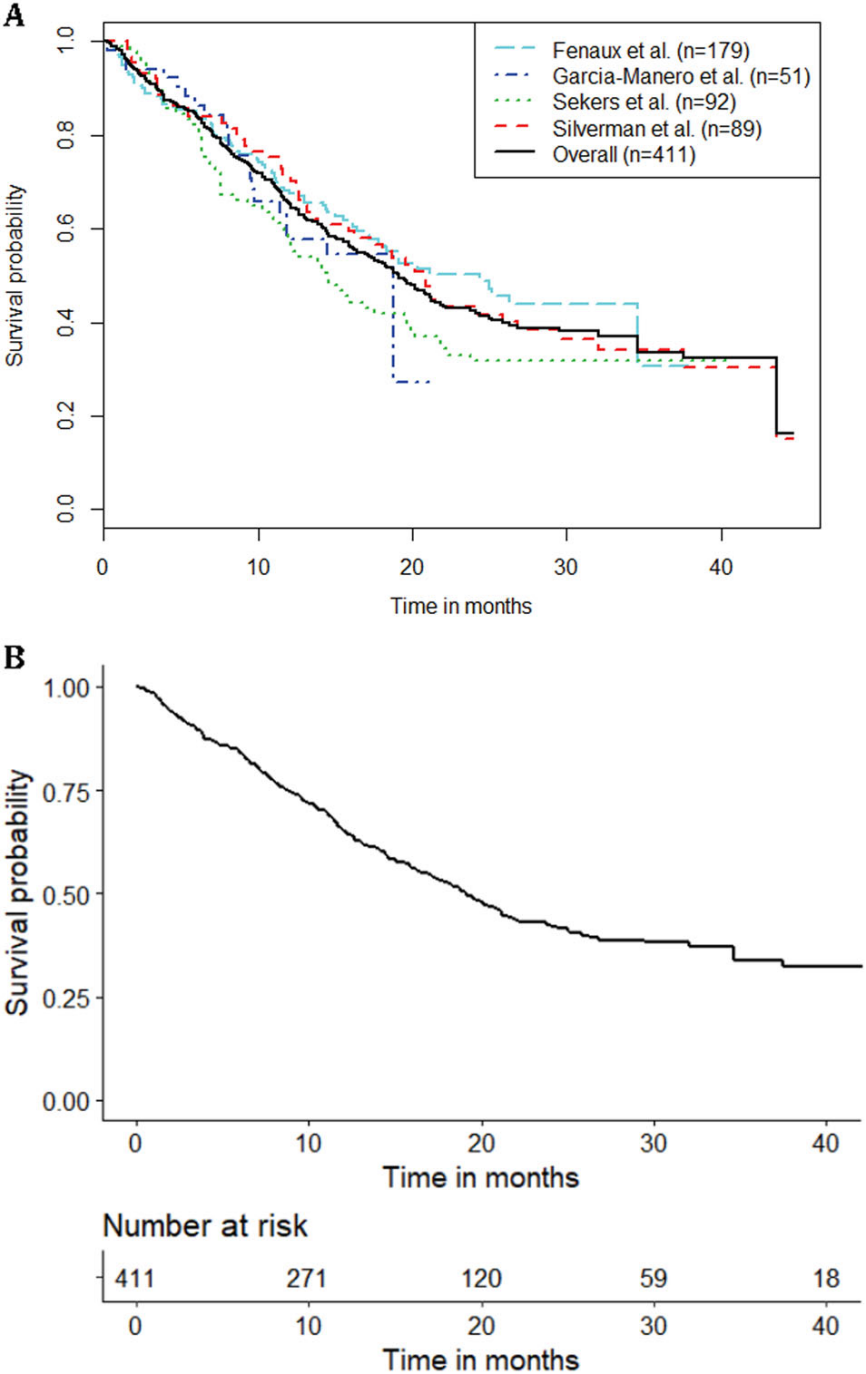
Overall Survival with azacitidine therapy. **a** KM estimates of OS for the azacitidine arm of each of the four articles, as well as the overall curve when all the individual level patient data are combined into one group (*n* = 411). **b** KM estimates of and numbers at risk for the pooled individual patient level data for patients who received azacitidine monotherapy in clinical trials

We found four published articles that fit the research criteria^1,12–14^ (Table 1). The baseline characteristics of patients who received azacitidine monotherapy in these trials are relatively comparable in terms of age, Eastern Cooperative Oncology Group Performance Status (ECOG PS), and distribution of risk status as reflected by the International Prognostic Scoring System (IPSS). When KM curves for the azacitidine arm of each of the four articles were pooled (Figs. 1a, b), median OS was 19.2 months (95% CI 16.9–21.8 months). KM estimates of OS for pooled data at 1 year was 65.4% (95% CI 60.8–70.3%), at 2 years was 42.4% (95% CI 37.3–48.3%), and at 3 years was 33.6% (95% CI 27.6–40.8%).

**Table 1:** Summary of articles included

Paper	Treatment Dosing and treatment schedule Median number of cycles	*N*	Age Median (range)	ECOG PS	IPSS	Median Time of follow-up in months (range)	Median OS in months (95% CI)
Fenaux et al. (AZA-001) ^1^	Azacitidine 75 mg/m^2^ for 7 days every 28 days 4 cycles (IQR 4–15)	179	69 (42–83)	0: 44% 1: 48% 2: 7% NA: 1%	Low: 0% Int-1: 3% Int-2: 43% High: 46%	21.1 (IQR 15–27)	24.5 (9.9–not reached)
Garcia-Manero et al. ^12^	Azacitidine + Placebo 75 mg/m^2^ for 7 days every 28 days Median number of cycles not shown: 69% received ≥ 5 cycles	51	69 (43–83)	0: 31% 1: 61% 2: 0% A: 8%	Low: 0% Int-1: 0% Int-2: 67% High: 33%	15	19
Sekeres et al. ^13^	Azacitidine 75 mg/m^2^ for 7 days every 28 days 6 cycles	92	69 (42–88)	0: 31% 1: 59% 2: 10% NA: 0%	Low: 3% Int-1: 28% Int-2: 45% High: 23%	23 (1–43)	15
Silverman et al.^14^	Azacitidine 75 mg/m^2^ for 7 days every 28 days Median number of cycles not shown, response was assessed after the fourth cycle	99	69 (31–92)	Not provided	Low: 2% Int-1: 26% Int-2: 11% High: 9%	Not provided	20 (16–26)

*ECOG PS* Eastern Cooperative Oncology Group Performance Status, *IPSS* International Prognostic Scoring System, *NA* not available, *IQR* interquartile range

In conclusion, pooled data from clinical trials with azacitidine monotherapy arms further support the real-life observation that the median OS of 24.5 months with azacitidine in AZA-001 trial reflects a substantial unexplained over-performance of azacitidine that is not related to the selective process of enrollment in clinical trials. A limitation of the study is that the data were pooled from different trials, which included disparate patient populations and used slightly different study designs.

One commonly cited reason for the underperformance of azacitidine compared with AZA-001 is the potential suboptimal use of azacitidine with regard to the administration schedule and persistence of therapy after a response is achieved^2^. Indeed, many community and some academic practices use alternative administration schedules to avoid weekend administration as demonstrated in the AVIDA registry study in which only 15% of MDS patients received azacitidine on the 7-day continuous administration schedule used in AZA-001^15^. However, both the French compassionate use program^5^ and the Spanish MDS registry study ^4^ used the 7-day consecutive schedule of azacitidine in most patients without approaching the OS reported in AZA-001. Even more, in the Spanish compassionate use program study^7^, there was no statistically significant difference in OS between patients receiving azacitidine on a 7-day consecutive schedule vs. on alternative schedules. The observed median OS in the other three randomized trials included in our analysis argue against an effect of the schedule (Table 1).

Due to different dynamics of response achievement in comparison with standard chemotherapy and the rarity of MDS, azacitidine is sometimes discontinued prematurely in patients before a minimum period of 6 months (which is recommended on the basis that 87% of patients who responded in AZA-001 achieved their response within the first 6 cycles) has passed^2^. For example, in the AVIDA registry patients received only a median of four cycles of azacitidine^15^ and in the Groupe Français des Myélodysplasies compassionate use program 17% of patients received <4 cycles of azacitidine^5^. However, in the SWOGS1117 study patients received a median of six cycles of azacitidine^13^ and in the phase II trial by Garcia-Manero et al. 69% of patients received ≥5 cycles of azacitidine^12^, still patients in both studies had significantly shorter OS compared with patients in the AZA-001 trial (Table 1).

Based on our study and a growing body of evidence, a median OS of 19 months (based on trial data of selected patients) and 13–16 months (based on real-life analyses) might be more realistic estimates for most patients with HR-MDS using azacitidine. Although individual patients have exceptionally long-lasting responses with azacitidine, such responses are uncommon. Indeed, a recent analysis from SEER Medicare data showed that the 5-year OS probability for patients with RAEB who were treated with hypomethylating agents was a dismal 4% (95% CI 2–6%)^16^. Additionally, it is important to keep in mind that only about half of HR-MDS patients treated with hypomethylating agents experience a response, and there are no reliable methods to predict whether patients will benefit from this therapy^2^. Even if patients are initially responding to hypomethylating agent therapy, once therapy fails, the median OS is only 4–6 months and no current therapy has been shown to add a survival benefit in that setting^2^. In this context, our analysis adds to the exiting evidence of the suboptimal performance of azacitidine and provides a rationale to strongly consider first-line enrollment into clinical trials or transplantation for HR-MDS patients rather than defaulting to the routine use of “standard-of-care” azacitidine monotherapy^2^.
